# Central and Obstructive Apneas in Heart Failure With Reduced, Mid-Range and Preserved Ejection Fraction

**DOI:** 10.3389/fcvm.2019.00125

**Published:** 2019-09-06

**Authors:** Chiara Borrelli, Francesco Gentile, Paolo Sciarrone, Gianluca Mirizzi, Giuseppe Vergaro, Nicolò Ghionzoli, Francesca Bramanti, Giovanni Iudice, Claudio Passino, Michele Emdin, Alberto Giannoni

**Affiliations:** ^1^Fondazione Toscana G. Monasterio, Pisa, Italy; ^2^Institute of Life Sciences, Scuola Superiore Sant'Anna, Pisa, Italy; ^3^Emergency Medicine Division, University of Pisa, Pisa, Italy

**Keywords:** heart failure, heart failure with reduced ejection fraction, heart failure with mid-range ejection fraction, heart failure with preserved ejection fraction, obstructive apneas, central apneas

## Abstract

**Background:** Although central apneas (CA) and obstructive apneas (OA) are highly prevalent in heart failure (HF), a comparison of apnea prevalence, predictors and clinical correlates in the whole HF spectrum, including HF with reduced ejection fraction (HFrEF), mid-range EF (HFmrEF) and preserved EF (HFpEF) has never been carried out so far.

**Materials and methods:** 700 HF patients were prospectively enrolled and then divided according to left ventricular EF (408 HFrEF, 117 HFmrEF, 175 HFpEF). All patients underwent a thorough evaluation including: 2D echocardiography; 24-h Holter-ECG monitoring; cardiopulmonary exercise testing; neuro-hormonal assessment and 24-h cardiorespiratory monitoring.

**Results:** In the whole population, prevalence of normal breathing (NB), CA and OA at daytime was 40, 51, and 9%, respectively, while at nighttime 15, 55, and 30%, respectively. When stratified according to left ventricular EF, CA prevalence decreased (daytime: 57 vs. 43 vs. 42%, *p* = 0.001; nighttime: 66 vs. 48 vs. 34%, *p* < 0.0001) from HFrEF to HFmrEF and HFpEF, while OA prevalence increased (daytime: 5 vs. 8 vs. 18%, *p* < 0.0001; nighttime 20 vs. 29 vs. 53%, *p* < 0.0001).

In HFrEF, male gender and body mass index (BMI) were independent predictors of both CA and OA at nighttime, while age, New York Heart Association functional class and diastolic dysfunction of daytime CA. In HFmrEF and HFpEF male gender and systolic pulmonary artery pressure were independent predictors of CA at daytime, while hypertension predicted nighttime OA in HFpEF patients; no predictor of nighttime CA was identified. When compared to patients with NB, those with CA had higher neuro-hormonal activation in all HF subgroups. Moreover, in the HFrEF subgroup, patients with CA were older, more comorbid and with greater hemodynamic impairment while, in the HFmrEF and HFpEF subgroups, they had higher left atrial volumes and more severe diastolic dysfunction, respectively. When compared to patients with NB, those with OA were older and more comorbid independently from background EF.

**Conclusions:** Across the whole spectrum of HF, CA prevalence increases and OA decreases as left ventricular systolic dysfunction progresses. Different predictors and specific clinical characteristics might help to identify patients at risk of developing CA or OA in different HF phenotypes.

## Introduction

Apneas, either in the form of obstructive apneas (OA) or central apneas (CA), are frequently observed in patients with heart failure and reduced ejection fraction (HFrEF, defined by a left ventricular ejection fraction—LVEF, <40%) (1). In this setting, several studies have defined the prevalence of apneas (2–5), their clinical significance (2, 6–8) and impact on outcome (2, 4, 6, 7, 9, 10), especially when present also during the daytime (2, 5, 6, 10).

In HFrEF patients, CA are present both at nighttime—with a prevalence range of 20–70% (1–3)—and at daytime—with a prevalence range of 16–60% (5, 6)—depending on the apnea-hypopnea index (AHI) threshold used and the severity of the HF population recruited. Predictors of CA in HFrEF patients identified in different studies were age, male gender, body mass index (BMI), LVEF, atrial fibrillation, awake partial pressure of carbon dioxide and increased natriuretic peptides (5, 11, 12). In HFrEF, CA have been associated with worse symptoms (1, 4, 6), reduced exercise tolerance and peak oxygen consumption (3, 5, 7), increased ventilatory inefficiency on effort, worse left ventricular (LV) systolic and diastolic function (2, 5), increased sympathetic activation and plasma levels of natriuretic peptides (2, 5) as well as increased incidence of atrial and ventricular arrhythmias (13, 14). Moreover, CA have been associated with an increased risk of mortality, both for HF progression and life-threating arrhythmias (2, 4, 6, 7, 9, 10), especially when CA are present also during the daytime (2, 5, 6, 10).

On the other hand, in HFrEF patients, OA are present mainly at nighttime, with a prevalence range of 13–40% (2, 15). Predictors of OA in HFrEF are BMI and concomitant hypertension (11, 16), while, clinically, OA have been associated with higher LV and right ventricular function and exercise tolerance when compared to HFrEF patients with CA (2, 16). Finally, OA also carry prognostic significance in patients with HFrEF, even though mortality risk seems lower than that experienced by patients with CA (2, 17).

The epidemiological and clinical relevance of OA and CA in patients with HF and mid-range ejection fraction (HFmrEF, defined by LVEF between 40 and 49%) (1) and HF and preserved ejection fraction (HFpEF, defined by LVEF ≥ 50%) (1) have been poorly addressed so far. In a study on 244 inpatients with HFpEF (defined with an LVEF > 55%), OA were found in 39.8% and were associated with higher rate of comorbidities, while CA were observed in 29.5% of patients and were associated with worse diastolic function, higher filling pressures, left atrial dimensions and natriuretic peptides levels (18). Results from another study, evaluating 115 outpatients with HF (38% of whom—*n* = 43—had either HFmrEF or HFpEF defined with by a LVEF ≥45%), reported a 62% prevalence of OA and 18% prevalence of CA (19), albeit no information on daytime apneas, CA/OA predictors and clinical correlates was provided.

Therefore, given the lack of knowledge in this field and hypothesizing a potential clinical impact of both OA and CA also in patients with HFmrEF and HFpEF the aim of this study was to investigate the prevalence, predictors and clinical significance of OA and CA across the whole spectrum of HF.

## Methods

### Patient Population

In the current study, we incorporated 525 patients with systolic HF (LVEF <50%) of a previously published study (2). Those patients were then divided according to 2016 ESC HF guidelines (1) based on the underlying LVEF (408 patients were classified as HFrEF and 117 patients were classified as HFmrEF).

In the same period (from January 2006 to December 2013), a population of 175 consecutive patients with HFpEF was also enrolled. For both patients with HFmrEF and HFpEF the presence of additional guidelines-recommended criteria (signs/symptoms of HF, elevated plasmatic concentrations of natriuretic peptides or relevant structural heart disease or diastolic dysfunction) was also considered (1).

Exclusion criteria were: acute coronary syndromes or episodes of acute HF within 3 months; severe pulmonary, renal or neurological disease; therapy with drugs affecting the respiratory drive such as morphine or derivatives, theophylline, oxygen, benzodiazepines, acetazolamide, or treatment with continuous positive airway pressure or servoventilation. Written informed consent was obtained from each patient before enrolment, and the study was approved by the Institutional Ethics Committee and conducted in accordance with Declaration of Helsinki of the World Medical Association.

### Study Design

All patients underwent a comprehensive evaluation (2, 20) with: 2-dimensional transthoracic echocardiography (IE33 ultrasound machine, Philips Medical Systems, Palo Alto, California), 24-h ECG Holter recording (Elamedical, Paris, France), symptom-limited cardiopulmonary exercise testing (VMAX, Sensormedics, Conshohocken, Pennsylvania, US) and biohumoral characterization, including assessment of plasma levels of norepinephrine, aldosterone, renin activity and N-terminal fraction of pro–B-type natriuretic peptide (NT-proBNP). Finally, all patients also underwent 24-h cardiorespiratory polygraphic recording for screening of CA/OA, as previously reported (2) and as explained below. All examinations were performed within 3-days in condition of clinical stability and without altering HF related treatments.

### 24-h Cardiorespiratory Polygraphic Recording

All patients underwent a 24-h continuous polygraphic recording to identify respiratory events and to classify them as central or obstructive. According to the latest guidelines of the American Academy of Sleep Medicine (21), three essential signals were incorporated: (1) nasal airflow; (2) chest and abdominal respiratory movements by inductance plethysmography; (3) oxygen saturation (SaO_2_) (Somté PSG2; Compumedics).

An apnea was defined as a cessation of breathing lasting >10 s; presence or absence of thoracic and abdominal movements allowed distinction between central and obstructive events, respectively. A hypopnea was defined as a reduction in airflow >50% of normal, lasting >10 s, with a SaO_2_ reduction ≥4% (21).

The presence/absence of a respiratory disturbance was established by means of the AHI (22). CA were diagnosed in presence of an AHI ≥5 events/h, with >50% of apneic events being central, whereas a diagnosis of OA was made in presence of an AHI ≥5 events/h, with >50% of apneic events being obstructive (22). CA/OA severity was then classified as being: mild with an AHI ≥ 5 and <15 events/h, moderate-severe with an AHI ≥15 and <30 events/h, and severe with an AHI ≥30 events/h. Given the potential misclassification of hypopneas without the use of esophageal pressure transducer or diaphragmatic electromyography and the poor reliability of indirect algorithms (23), we considered hypopneas to follow the distribution of the majority of the apneic events (24). Nonetheless, we also performed an analysis exclusively based on the apneic events using the central apnea index (number of CA per hour, CAI) and the obstructive apnea index (number of OA per hour, OAI). Finally, we also evaluated the burden of desaturation with the minimum SaO_2_ and the time spent with a SaO_2_ <90% (T90).

The AHI, CAI, and OAI were computed over the whole 24-h recording, at night (10 p.m. to 6:59 a.m.) and during daytime (7 a.m. to 9:59 p.m.), as previously reported (2). Data analysis was performed by experienced sleep technicians (G.I. and F.B.), and then controlled by a physician with specific relevant clinical and research experience in the field (A.G., C.B., M.E., or C.P.).

### Statistical Analysis

Statistical analysis was performed using SPSS 21.0 program (1989–2012, LEAD technologies Inc., USA). Values were presented as mean ± standard deviation (SD) for variables with normal distribution, median and interquartile range (IR) for variables with skewed distribution and as percentage for categorical data. Variables with a skewed distribution were first log transformed before regression analysis. To address predictors of OA/CA, univariate logistic regression analysis was first performed. Statistically significant variables (*p* < 0.05) were than manually inserted in a multivariable logistic regression analysis to identify independent predictors of OA/CA. For continuous variables, differences between 2 groups were evaluated through the independent student *T*-test and Mann-Whitney U test, while differences among >2 groups were evaluated through ANOVA or Kruskal–Wallis one-way analysis of variance, with Bonferroni *post-hoc* correction. For categorical variables, differences were analyzed with the Chi-Square with Yates correction or Fisher's exact test, as appropriate. A two-tailed *p*-value <0.05 was considered statistically significant.

## Results

### Patient Population

The clinical characteristics of the general population and the different HF subgroups (700 patients, 408 with HFrEF, 117 with HFmrEF and 175 with HFpEF) are summarized in Table 1.

**Table 1:** Characteristics of general population.

	**All patients *N* = 700**	**HFrEF *N* = 408**	**HFmrEF *N* = 117**	**HFpEF *N* = 175**
Age (years)	66.8 ± 12.4	65.3 ± 12.9	67.3 ± 11.9	71.4 ± 9.8^d^
Males (%)	74	78	76	65^c^
BMI (kg/m^2^)	28.3 ± 5.4	27.2 ± 5.1	28.6 ± 5.2	31.4 ± 8.7^d^^,^ ^f^
NYHA I-II/III-IV (%)	67/33	63/37	76/24	70/30
DCM (%)	43	54	51	12^d^^,^ ^f^
ICM(%)	38	44	46	18^d^^,^ ^f^
Other etiology (%)	19	2	3	70^d^^,^ ^f^
**Comorbidities**				
Atrial fibrillation (%)	29	24	31	40^c^
Systemic hypertension (%)	59	50	63	75^d^
Diabetes mellitus (%)	31	30	28	35
COPD (%)	18	15	19	26^c^
Anemia (%)	34	31	31	44^c^
Hb (g/dL)	13.3 ± 1.8	13.4 ± 1.7	13.3 ± 1.8	12.8 ± 1.8^d^^,^ ^e^
Creatinine (mg/dL)	1.2 ± 0.5	1.2 ± 0.5	1.2 ± 0.6	1.1 ± 0.5^c^
eGFR (mL/min/1.73m^2^)	64 (49–81)	62 (48–78)	67 (49–87)	67 (51–83)
TSH (μUI/mL)	1.8 (1.1–2.8)	1.8 (1.1–2.8)	1.5 (0.9–2.6)	2.0 (1.3–3.0)
C-reactive protein (mg/dL)	0.3 (0.1–0.8)	0.3 (0.1–0.8)	0.3 (0.1–0.8)	0.3 (0.2–1.0)
**Echocardiography**				
LVEF (%)	37.9 ± 13.9	28.3 ± 6.8	43.1 ± 2.8^b^	57.3 ± 6.8^d^^,^ ^f^
LA volume (mL/m^2^)	40.3 ± 14.2	40.6 ± 12.8	36.6 ± 11.3^a^	39.1 ± 11.0
Diastolic dysfunction II-III (%)	29	48	30^a^	21^d^
Moderate-severe MR (%)	43	48	28^a^	40
TAPSE (mm)	18.5 ± 5.0	17.7 ± 4.9	18.7 ± 5.1	20.0 ± 4.9^d^
sPAP (mmHg)	41.8 ± 12.6	43.3 ± 12.5	40.0 ± 12.6^a^	39.7 ± 12.3^c^
**Neurohormonal activation**				
Hs-Troponin T (ng/L)	20.4 (12.3–42.8)	20.3 (12.7–34.9)	38.8 (19.7–62.6)	20.3 (12.2–46.6)
NT-proBNP (pg/mL)	1,214 (459–2,938)	1,569 (669–3,715)	657 (219–2,127)^b^	809 (340–1,876)^d^
Norepinephrine (pg/mL)	432 (284–634)	471 (300–657)	385 (254–512)^a^	397 (246–669)
Aldosterone (pg/mL)	123 (74–193)	127 (79–198)	113 (74–185)	110 (48–188)
PRA (ng/mL/h)	0.9 (0.2–2.6)	1.1 (0.3–3.2)	0.5 (0.2–1.4)^b^	0.3 (0.2–1.1)^d^
Peak VO_2_ (%)	57.7 ± 17.1	54.7 ± 16.4	66.1 ± 17.2^b^	60.8 ± 16.6^c^
VE/VCO_2_ slope	33.2 ± 8.1	33.8 ± 8.4	30.2 ± 6.7^a^	33.9 ± 5.5^e^
**Therapy**				
β-blockers (%)	89	96	96	68^d^^,^ ^f^
ACEi/ARB (%)	87	94	88	69^d^^,^ ^e^
MRA (%)	68	83	55^b^	41^d^
Furosemide (%)	47	48	40	62^c^^,^ ^e^
ICD/CRT-D (%)	22/19	35/26	6/4^b^	2/13^d^

*ACEi, angiotensin-converting enzyme inhibitors; ARB, angiotensin II receptor blockers; BMI, body mass index; COPD, chronic obstructive pulmonary disease; CRP, C-reactive protein; CRT-D, cardiac resynchronization therapy with defibrillator; DCM, dilated cardiomyopathy; eGFR, estimated glomerular filtration rate (with the Modification of Diet in Renal Disease-MDRD formula); Hb, hemoglobin; Hs, high sensitive; ICD, implantable cardioverter defibrillator; ICM, ischemic cardiomyopathy; LA, left atrium; LVEF, left ventricular ejection fraction; LVMI, left ventricular mass index; MR, mitral regurgitation; MRA, mineralocorticoid receptor antagonists; NT-proBNP, N-terminal fraction of pro–B-type natriuretic peptide; NYHA, New York Heart Association; sPAP, systolic pulmonary artery pressure; PRA, plasmatic renin activity; TAPSE, tricuspid annular plane systolic excursion; TSH, thyroid stimulating hormone; VE/VCO_2_, ventilation to carbon dioxide production ratio; VO_2_, oxygen consumption*.

*Values are expressed as mean ± standard deviation for variables with normal distribution, median (interquartile range) for variables with skewed distribution and percentages for categorical variables*.

a *p < 0.05 HFrEF vs. HFmrEF*.

b *p < 0.001 HFrEF vs. HFmrEF*.

c *p < 0.05 HFrEF vs. HFpEF*.

d *p < 0.001 HFrEF vs. HFpEF*.

e *p < 0.05 HFmrEF vs. HFpEF*.

f *p < 0.001 HFmrEF vs. HFpEF*.

Dilated cardiomyopathy was the most prevalent HF etiology in HFrEF and HFmrEF (*p* < 0.001 vs. HFpEF), while cardiomyopathy of other etiology (i.e., valvular disorders, amyloidosis, hypertrophic cardiomyopathy) was more prevalent in HFpEF (*p* < 0.001). Patients with HFpEF were older (*p* < 0.0001) and had more comorbidities than patients with HFrEF (namely: atrial fibrillation, hypertension, chronic obstructive pulmonary disease, anemia; all *p* < 0.05).

Patients with HFrEF had worse hemodynamic compromise compared to the other HF subgroups, as expressed by more severe diastolic dysfunction and increased pulmonary artery pressure (vs. both HFmrEF and HFpEF, all *p* < 0.05), greater left atrium (LA) volume and prevalence of moderate-severe mitral regurgitation (MR) (vs. HFmrEF, all *p* < 0.05), and worse right ventricular function (vs. HFpEF, *p* < 0.001).

Furthermore, patients with HFrEF had higher values of NT-proBNP and plasma renin activity when compared to both HFmrEF and HFpEF (both *p* < 0.001), and higher norepinephrine when compared to HFmrEF only (*p* = 0.02). Patients with HFrEF were more frequently treated with mineralocorticoid receptor antagonists and ICD/CRT-D than patients with HFmrEF and HFpEF (both *p* < 0.001), while patients with HFpEF were more often treated with loop diuretics than patients with HFrEF and HFmrEF (all *p* < 0.05).

### Distribution of Apneas During the 24-h Across the Whole HF Spectrum

**Figure 1:**
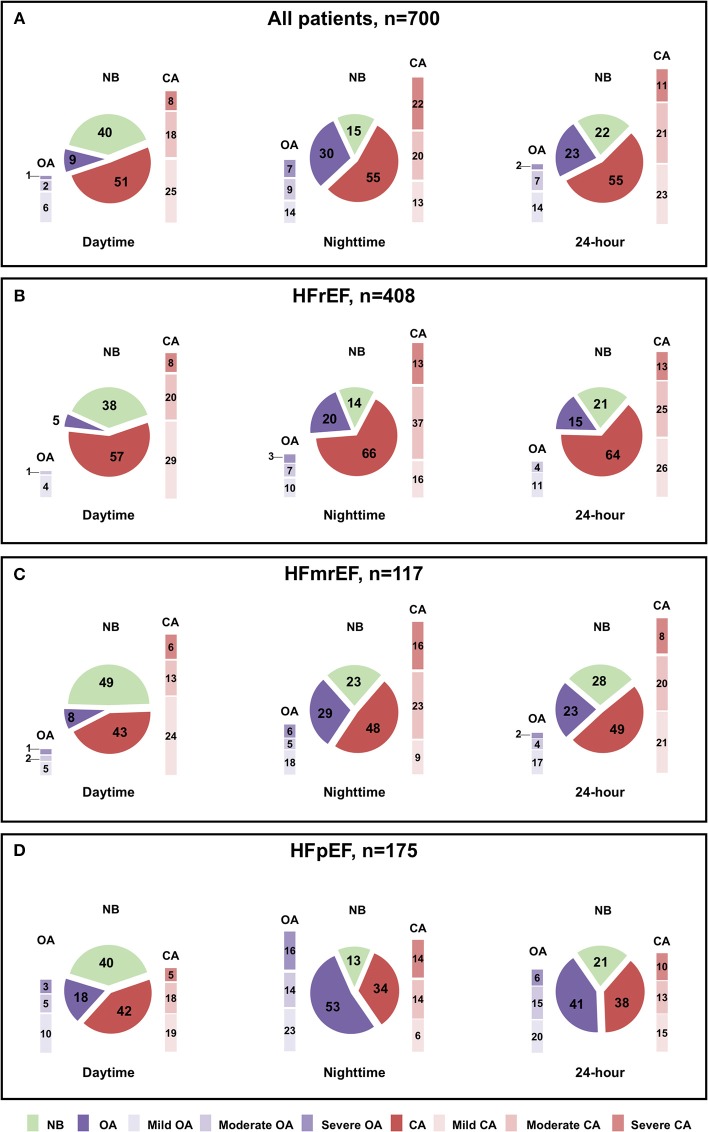
Prevalence of normal breathing (NB), central apneas (CA) and obstructive apneas (OA) during daytime, nighttime and 24-h, across the whole heart failure (HF) spectrum. **(A)** Prevalence of NB, CA and OA in the general population of HF patients during daytime, nighttime and 24-h. **(B)** Prevalence of NB, CA and OA in HF with reduced ejection fraction (HFrEF) during daytime, nighttime and 24-h. **(C)** Prevalence of NB, CA, and OA in HF with mid-range ejection fraction (HFmrEF) during daytime, nighttime and 24-h. **(D)** Prevalence of NB, CA and OA in HF with preserved ejection fraction (HFpEF) during daytime, nighttime and 24-h.

Prevalence rates of patients with NB, OA and CA during daytime, nighttime and 24-h in the whole population and in the three HF phenotypes are shown in Figure 1, while data on the AHI, CAI, OAI, minimal SaO_2_ and T90 are reported in Table 2.

**Table 2:** 24-h cardiorespiratory monitoring across the whole HF spectrum.

	**All patients *N* = 700**	**HFrEF *N* = 408**	**HFmrEF *N* = 117**	**HFpEF *N* = 175**
Diurnal apnea prevalence (%)	60	62	51	60
Nocturnal apnea prevalence (%)	85	86	77	87
24-h apnea prevalence (%)	78	79	72	79
Diurnal CA prevalence (%)	51	57	43^a^	42^b^
Nocturnal CA prevalence (%)	55	66	48^a^	34^b^^,^ ^c^
24-h CA prevalence (%)	55	64	49^a^	38^b^
Diurnal OA prevalence (%)	9	5	8	18^b^^,^ ^c^
Nocturnal OA prevalence (%)	30	20	29	53^b^^,^ ^c^
24-h OA prevalence (%)	23	15	23	41^b^^,^ ^c^
Diurnal AHI (events/h)	8 (2–17)	9 (2–17)	6 (2–14)	8 (3–19)
Nocturnal AHI (events/h)	19 (8–33)	20 (8–33)	16 (6–29)	21 (11–35)
24-h AHI (events/h)	13 (5–23)	13 (5–22)	4 (10–20)	13 (6–25)
Diurnal CAI (events/h)	1 (0–5)	1 (0–7)	0 (0–4)^a^	0 (0–3)^b^
Nocturnal CAI (events/h)	2 (0–11)	3 (0–16)	2 (0–9)^a^	1 (0–5)^b^
24-h CAI (events/h)	2 (0–8)	2 (0–11)	1 (0–6)^a^	1 (0–4)^b^
Diurnal OAI (events/h)	0 (0–1)	0 (0–0)	0 (0–1)	0 (0–1)^b^^,^ ^c^
Nocturnal OAI (events/h)	0 (0–2)	0 (0–1)	0 (0–3)^a^	1 (0–5)^b^^,^ ^c^
24-h OAI (events/h)	0 (0–2)	0 (0–0)	0 (0–2)^a^	1 (0–3)^b^^,^ ^c^
SaO_2_ min (%)	85 (80–88)	86 (81–89)	85 (80–89)	82 (78–86)
T90 (min)	8 (3–16)	6 (2–13)	8 (2–17)	12 (5–18)

*AHI, apnea-hypopnea index; CA, central apneas; CAI, central apnea index; OA, obstructive apneas; OAI, obstructive apnea index; T90, time spent with an oxygen saturation below 90%*.

*Values are expressed as median (interquartile range) for variables with skewed distribution and percentages for categorical variables*.

a *p < 0.05 HFrEF vs. HFmrEF*.

b *p < 0.001 HFrEF vs. HFpEF*.

c *p < 0.05 HFmrEF vs. HFpEF*.

In the whole population, using an AHI cut-off ≥5 events/h, the prevalence of NB, CA, and OA was 40, 51, and 9% at daytime and 15, 55, and 30% at nighttime, respectively.

When stratified according to LVEF, CA were more prevalent in HFrEF than HFmrEF and HFpEF both at daytime (57 vs. 43 vs. 42%; comparison between groups, *p* = 0.001) and nighttime (66 vs. 48 vs. 34%; comparison between groups *p* < 0.0001), while prevalence of OA was higher in HFpEF than in HFrEF and HFmrEF, both at daytime (5 vs. 8 vs. 18%; comparison between groups *p* < 0.0001) and nighttime (20 vs. 29 vs. 53%; comparison between groups, *p* < 0.0001).

Using an AHI cut-off for moderate-severe apneas ≥15 events/h, daytime CA prevalence was similar between the three groups (28 vs. 19 vs. 23%; *p* between groups = 0.09), while nighttime CA was still higher in HFrEF (50 vs. 39 vs. 28%; *p* between groups <0.0001). Conversely, moderate-severe OA were less prevalent in HFrEF and HFmrEF than in HFpEF both at daytime (1 vs. 3 vs. 8%; *p* between groups <0.001) and nighttime (10 vs. 11 vs. 30%; *p* between groups <0.0001).

### Predictors of Apneas Across the Whole Spectrum of HF

#### Predictors of Central Apneas

Univariarable and multivariable predictors of CA at nighttime are shown in Table 3. In the whole population only male gender and LA volume remained independent predictors of nighttime CA (male gender: OR 10.95 CI 2.87–41.78, *p* < 0.001; LA volume OR 1.09 CI 1.01–1.19, *p* = 0.04). When stratified according to LVEF, in the HFrEF population male gender and BMI remained as independent predictors of CA at nighttime (male gender: OR 2.99 CI 1.18–7.63, *p* = 0.02; BMI: OR 1.14 CI 1.02–1.26, *p* = 0.02), while no predictors were found in HFmrEF and HFpEF populations.

**Table 3:** Predictors of central apneas at nighttime.

	**Univariate analysis**			**Multivariate analysis**		
	**OR**	**CI**	***P*-value**	**OR**	**CI**	***P*-value**
**In the whole population**						
Age	1.04	1.03–1.06	<0.001	-	-	*-*
Male gender	4.09	2.58–6.49	<0.001	10.95	2.87–41.78	<0.001
NYHA class	1.51	1.13–2.04	0.006	-	-	*-*
ICM	2.17	1.34–3.50	0.001	-	-	*-*
Systemic hypertension	1.80	1.16–2.79	0.009	-	-	*-*
Hb	1.16	1.02–1.32	0.020	-	-	*-*
C-reactive protein	1.23	1.03–1.46	0.020	-	-	*-*
LVEF	0.97	0.95–0.99	<0.001	-	-	*-*
LVMI	1.01	1.00–1.01	0.010	-	-	*-*
LA volume	1.07	1.04–1.10	<0.001	1.09	1.01–1.19	0.040
Diastolic dysfunction II-III	3.23	1.82–5.73	<0.001	-	-	*-*
Severe MR	2.56	1.13–5.79	0.020	-	-	*-*
TAPSE	0.93	0.89–0.98	0.005	-	-	*-*
sPAP	1.05	1.02–1.08	0.001	-	-	*-*
NT-proBNP	1.52	1.29–1.80	<0.001	-	-	*-*
Norepinephrine	2.31	1.60–3.33	<0.001	-	-	*-*
ICD	1.85	1.06–3.22	0.03	-	-	*-*
**In the HFrEF population**						
Age	1.05	1.03–1.07	<0.001	**-**	**-**	**-**
Male gender	4.32	2.36–7.88	<0.001	2.99	1.18–7.63	0.020
BMI	1.10	1.02–1.18	0.009	1.14	1.02–1.26	0.020
NYHA class	1.60	1.09–2.35	0.020	-	-	*-*
ICM	3.27	1.68–6.32	<0.001	-	-	*-*
Atrial fibrillation	2.45	1.12–5.46	0.030	-	-	*-*
Systemic hypertension	2.29	1.26–4.17	0.007	-	-	*-*
Creatinine	2.65	1.20–5.81	0.020	-	-	*-*
eGFR	0.99	0.98–1.00	0.020	-	-	*-*
LVEF	0.94	0.90–0.98	0.008	-	-	*-*
LVMI	1.01	1.00–1.02	0.010	-	-	*-*
LA volume	1.07	1.03–1.12	<0.001	-	-	*-*
Diastolic dysfunction II-III	3.30	1.60–6.83	0.001	-	-	*-*
**In the HFmrEF population**						
Male gender	6.60	2.01–21.44	0.002	-	-	*-*
LA volume	1.10	1.01–1.20	0.020	-	-	*-*
sPAP	1.12	1.01–1.23	0.030	-	-	−
**In the HFpEF population**						
Age	1.07	1.00–1.14	0.040	-	-	*-*
NT-proBNP	1.70	1.12–2.58	0.010	-	-	*-*
Norepinephrine	4.84	1.31–17.83	0.020	-	-	*-*

*BMI, body mass index; CI, confidence interval; eGFR, estimated glomerular filtration rate (with the Modification of Diet in Renal Disease-MDRD formula); Hb, hemoglobin; ICD, implantable cardioverter defibrillator; ICM, ischemic cardiomyopathy; LA, left atrium; LVEF, left ventricular ejection fraction; LVMI, left ventricular mass index; MR, mitral regurgitation; NT-proBNP, N-terminal fraction of pro–B-type natriuretic peptide; NYHA, New York Heart Association; OR, odds ratio; sPAP, systolic pulmonary artery pressure; TAPSE, tricuspid annular plane systolic excursion*.

Univariable and multivariable predictors of CA at daytime are shown in Table 4. In the whole population, age (OR 1.04 CI 1.01–1.08, *p* = 0.02), male gender (OR 5.19 CI 2.42–11.14, *p* < 0.0001) and LA volume (OR 1.04 CI 1.01–1.08, *p* = 0.03) were found to be independent predictors of daytime CA. In the HFrEF population age (OR 1.09 CI 1.03–1.16, p = 0.04), functional NYHA class (OR 2.28 CI 1.08–4.38, *p* = 0.03) and grade II-III diastolic dysfunction (OR 4.26 CI 1.16–15.69, *p* = 0.03) remained as independent predictors, while sPAP (OR 1.07 CI 0.99-1.15, *p* = 0.04) was the only independent predictor in HFmrEF. Conversely, no predictor was identified in the HFpEF population.

**Table 4:** Predictors of central apneas at daytime.

	**Univariate analysis**			**Multivariate analysis**		
	**OR**	**CI**	***P*-value**	**OR**	**CI**	***P*-value**
**In the whole population**						
Age	1.03	1.02–1.05	<0.0001	1.04	1.01–1.08	0.020
Male gender	2.59	1.81–3.69	<0.0001	5.19	2.42–11.14	<0.0001
NYHA class	1.51	1.23–1.85	<0.0001	-	-	*-*
ICM	1.72	1.23–2.41	0.002	-	-	*-*
Systemic hypertension	1.41	1.02–1.95	0.039	-	-	*-*
Diabetes mellitus	1.39	1.01–1.93	0.048	-	-	*-*
Creatinine	1.70	1.18–2.46	0.004	-	-	*-*
eGFR	0.99	0.98–0.99	0.001	-	-	*-*
LVEF	0.98	0.97–0.99	<0.0001	-	-	*-*
LVMI	1.01	1.00–1.01	0.004	-	-	*-*
LA volume	1.04	1.02–1.06	<0.0001	1.04	1.01–1.08	0.030
Diastolic dysfunction II-III	3.40	2.25–5.14	<0.0001	-	-	*-*
Moderate-severe MR	1.71	1.01–2.89	0.044	-	-	*-*
sPAP	1.02	1.00–1.04	0.033	-	-	*-*
**In the HFrEF population**						
Age	1.05	1.04–1.07	<0.0001	1.09	1.03–1.16	0.040
Male gender	2.34	1.45–3.79	0.001	-	-	*-*
BMI	1.05	1.00–1.10	0.031	-	-	*-*
NYHA class	1.77	1.33–2.36	<0.0001	2.28	1.08–4.38	0.030
ICM	2.18	1.42–3.36	<0.0001	-	-	*-*
Atrial fibrillation	1.88	1.14–3.13	0.014	-	-	*-*
Diabetes mellitus	1.59	0.99–2.54	0.050	-	-	*-*
Creatinine	2.59	1.51–4.45	0.001	-	-	*-*
eGFR	0.98	0.97–0.99	<0.001	-	-	*-*
C-reactive protein	1.21	1.02–1.43	0.025	-	-	*-*
LVEF	0.96	0.93–0.99	0.014	-	-	*-*
LA volume	1.06	1.03–1.09	<0.001	-	-	*-*
Diastolic dysfunction II-III	4.39	2.60–7.41	<0.001	4.26	1.16–15.69	0.030
sPAP	1.03	1.01–1.05	0.012	-	-	*-*
β-blockers	3.17	1.04–9.66	0.040	-	-	*-*
**In the HFmrEF population**						
Male gender	2.65	0.99–7.07	0.040	-	-	*-*
LVMI	1.02	1.00–1.04	0.015	-	-	*-*
sPAP	1.06	1.00–1.13	0.044	1.07	0.99–1.15	0.040
**In the HFpEF population**						
Male gender	3.00	1.51–6.09	0.002	-	-	*-*

*BMI, body mass index; CI, confidence interval; eGFR, estimated glomerular filtration rate (with the Modification of Diet in Renal Disease-MDRD formula); Hb, hemoglobin; ICM, ischemic cardiomyopathy; LA, left atrium; LVEF, left ventricular ejection fraction; LVMI, left ventricular mass index; MR, mitral regurgitation; NYHA, New York Heart Association; OR, odds ratio; sPAP, systolic pulmonary artery pressure; TAPSE, tricuspid annular plane systolic excursion*.

#### Predictors of Obstructive Apneas

Considering the low prevalence of OA at daytime, the analysis of OA predictors was limited at the nighttime period.

Univariable and multivariable predictors of OA at nighttime are shown in Table 5. In the whole population, at multivariable logistic regression analysis, age (OR 1.04 CI 1.01–1.08, *p* = 0.03), male gender (OR 7.11 CI 3.07–16.50, *p* < 0.001) and BMI (OR 1.16 CI 1.06–1.27, *p* = 0.001) resulted independent predictors of OA at nighttime.

**Table 5:** Predictors of obstructive apneas at nighttime.

	**Univariate analysis**			**Univariate analysis**		
	**OR**	**CI**	***P*-value**	**OR**	**CI**	***P*-value**
**In the whole population**						
Age	1.05	1.03–1.07	<0.001	1.04	1.01–1.08	0.030
Male gender	2.64	1.61–4.33	<0.001	7.11	3.07–16.50	<0.001
BMI	1.15	1.09–1.21	<0.001	1.16	1.06–1.27	0.001
NYHA class	1.45	1.04–2.00	0.030	-	-	*-*
Systemic hypertension	2.85	1.75–4.65	<0.001	-	-	*-*
COPD	2.00	1.03–3.93	0.040	-	-	*-*
LVEF	1.03	1.01–1.05	0.002	-	-	*-*
LA volume	1.03	1.01–1.07	0.020	-	-	*-*
**In the HFrEF population**						
Age	1.04	1.01–1.06	0.004	-	-	*-*
Male gender	3.73	1.76–7.92	0.001	3.53	1.18–10.54	0.030
BMI	1.13	1.05–1.22	0.001	1.16	1.03–1.30	0.020
NYHA class	1.60	1.02–2.51	0.040	-	-	*-*
ICM	2.98	1.40–6.35	0.005	-	-	*-*
Systemic hypertension	2.52	1.24–5.11	0.010	-	-	*-*
LA volume	1.05	1.01–1.09	0.030	-	-	*-*
**In the HFmrEF population**						
Age	1.06	1.01–1.11	0.030	-	-	*-*
**In the HFpEF population**						
BMI	1.20	1.07–1.35	0.003	-	-	*-*
Systemic hypertension	2.99	1.14–7.87	0.030	2.84	1.07–7.57	0.040

*BMI, body mass index; CI, confidence interval; COPD, chronic obstructive pulmonary disease; ICM, ischemic cardiomyopathy; LA, left atrium; LVEF, left ventricular ejection fraction; NYHA, New York Heart Association; OR, odds ratio*.

Similarly, in the HFrEF population, at multivariable analysis, male gender (OR 3.53 CI 1.18–10.54, *p* = 0.03) and BMI (OR 1.16 CI 1.03–1.30, *p* = 0.02) remained the only independent predictors of OA at nighttime, while systemic hypertension in HFpEF (OR 2.84 CI 1.07–7.57, *p* = 0.04). On the contrary, no independent predictor of nighttime OA was found in the HFmrEF population.

### Clinical Correlates of Apneas Across the Whole Spectrum of HF

Clinical correlates of CA are summarized in Table 6 and Supplementary Tables 1–3.

**Table 6:** Clinical comparison between nighttime only and combined daytime and nighttime moderate-severe CA across the whole HF spectrum.

	**HFrEF**		**HFmrEF**		**HFpEF**	
	**N-CA**	**DN-CA**	**N-CA**	**DN-CA**	**N-CA**	**DN-CA**
%	32	27	25	19	29	29
Age (years)	65.3 ± 11.3	69.4 ± 11.0^a^	68.1 ± 13.3	70.2 ± 9.3	73.0 ± 8.4	71.0 ± 9.9
Males (%)	78	91^a^	76	96	75	82
BMI (kg/m^2^)	28.2 ± 5.2	28.0 ± 5.2	27.7 ± 4.7	28.1 ± 3.6	31.4 ± 6.2	33.2 ± 13.7
NYHA class I-II/III-IV (%)	67/33	48/52^a^	85/15	73/27	70/30	68/32
DCM (%)	49	51	55	36	8	12
ICM (%)	50	49	41	59	16	16
Other etiology (%)	1	0	3	5	76	73
**Comorbidities**						
Atrial fibrillation (%)	26	31	41	33	35	45
Systemic hypertension (%)	54	56	59	68	84	77
Diabetes mellitus (%)	31	39	14	36	41	43
COPD (%)	16	12	14	28	12	31^a^
Anemia (%)	26	25	24	32	49	48
Hb (g/dL)	13.5 ± 1.6	13.8 ± 1.8	13.6 ± 1.8	13.2 ± 2.1	12.6 ± 1.6	13.1 ± 2.0
Creatinine (mg/dL)	1.2 ± 0.6	1.4 ± 0.6^a^	1.2 ± 0.7	1.2 ± 0.9	1.1 ± 0.3	1.1 ± 0.4
eGFR (mL/min/1.73 m^2^)	60 (52–81)	57 (42–70)^a^	68 (50–88)	67 (47–82)	65 (51–89)	70 (55–80)
TSH (μUI/mL)	1.9 (1.2–2.8)	1.7 (1.1–2.8)	1.9 (1.2–2.6)	1.7 (0.9–2.2)	2.0 (1.6–3.1)	2.1 (1.4–2.6)
C-reactive protein (mg/dL)	0.3 (0.1–0.7)	0.4 (0.1–0.9)	0.4 (0.1–0.9)	0.2 (0.1–0.8)	0.3 (0.2–1.0)	0.4 (0.2–2.2)
**Echocardiography**						
LVEF (%)	28.3 ± 6.9	27.3 ± 6.8	42.9 ± 2.7	42.7 ± 2.5	56.2 ± 5.3	57.3 ± 6.0
LA volume (mL/m^2^)	42.2 ± 13.6	44.1 ± 13.0	39.5 ± 13.3	42.1 ± 10.5	40.0 ± 10.1	39.9 ± 12.8
Diastolic dysfunction II-III (%)	44	70^a^	54	26	30	20
Moderate-severe MR (%)	46	56	48	29	45	31
TAPSE (mm)	18.4 ± 5.0	16.9 ± 4.9	17.4 ± 5.4	18.4 ± 4.6	19.8 ± 4.6	20.3 ± 4.9
sPAP (mmHg)	43.9 ± 14.4	46.2 ± 12.2	44.9 ± 15.8	40.2 ± 12.9	39.1 ± 11.7	38.1 ± 11.4
**Neurohormonal activation and excercise tolerance**						
Hs-Troponin T (ng/L)	23 (11–35)	27 (13–47)	53 (12–185)	32 (18–40)	20 (13–54)	33 (13–54)
NT-proBNP (pg/mL)	1,230 (586–3,400)	2,365 (1,183–6,064)^b^	2,013 (214–4,033)	910 (450–1,505)	1,072 (395–1,889)	868 (338–2,089)
Norepinephrine (pg/mL)	449 (299–644)	552 (385–790)^a^	420 (314–483)	470 (261–662)	404 (261–476)	669 (373–1,214)^a^
Aldosterone (pg/mL)	124 (75–189)	106 (69–194)	129 (83–227)	91 (62–197)	86 (42–148)	179 (151–209)
PRA (ng/mL/h)	0.9 (0.2–4.0)	1.1 (0.3–2.5)	0.7 (0.3–1.7)	0.2 (0.1–1.2)	0.3 (0.2–1.0)	0.3 (0.2–1.2)
Peak VO_2_ (%)	59.2 ± 17.8	54.3 ± 13.5	64.9 ± 23.1	67.2 ± 12.9	60.7 ± 15.7	56.9 ± 13.1
VO_2_/Kg/min (mL/Kg/min)	14.7 ± 4.6	13.1 ± 3.6	16.9 ± 6.5	16.4 ± 4.4	12.8 ± 9.6	14.2 ± 3.8
VE/VCO_2_ slope	33.5 ± 7.1	36.6 ± 9.1	30.4 ± 7.3	31.2 ± 5.3	34.3 ± 6.3	32.5 ± 5.6
SaO_2_ min (%)	83.0 ± 9.4	80.1 ± 10.2^a^	82.3 ± 6.6	82.0 ± 8.5	81.1 ± 5.9	78.3 ± 7.2
T90 (min)	5 (2–11)	11 (5–22)^b^	10 (4–14)	9 (1–25)	5 (12–17)	17 (9–21)^a^
**Therapy**						
β-blockers (%)	97	98	93	100	74	67
ACEi/ARB (%)	93	92	86	91	82	57^a^
MRA (%)	84	82	55	50	46	41
Furosemide (%)	51	45	52	41	67	58
ICD/CRT-D (%)	62	61	8	10	14	20

a *p < 0.05 N-CA vs. DN-CA*.

b *p < 0.001 N-CA vs. DN-CA*.

*Apneas were defined as moderate-severe (AHI^≥^15 events/h). Patients were classified according to the presence of only nighttime apneas (N-CA) or the presence of both nighttime and daytime apneas (DN-CA).

*ACEi, angiotensin-converting enzyme inhibitors; ARB, angiotensin II receptor blockers; BMI, body mass index; COPD, chronic obstructive pulmonary disease; CRT-D, cardiac resynchronization therapy with defibrillator; DCM, dilated cardiomyopathy; eGFR, estimated glomerular filtration rate (with the Modification of Diet in Renal Disease-MDRD formula); Hb, hemoglobin; Hs, high sensitive; ICD, implantable cardioverter defibrillator; ICM, ischemic cardiomyopathy; LA, left atrium; LVEF, left ventricular ejection fraction; LVMI, left ventricular mass index; MR, mitral regurgitation; MRA, mineralocorticoid receptor antagonists; NT-proBNP, N-terminal fraction of pro–B-type natriuretic peptide; NYHA, New York Heart Association; PRA, plasmatic renin activity; SaO_2_:oxygen saturation; sPAP, systolic pulmonary artery pressure; T90, time spent with an oxygen saturation below 90%; TAPSE, tricuspid annular plane systolic excursion; TSH, thyroid stimulating hormone; VE/VCO_2_, ventilation to carbon dioxide production ratio; VO_2_, oxygen consumption*.

Compared to NB, patients with HFrEF and both nighttime (Supplementary Table 1) and daytime (Supplementary Table 2) CA had more frequently atrial fibrillation, higher LV filling pressures (as expressed by worse diastolic dysfunction and greater LA volume (all *p* < 0.001) and greater values of NT-proBNP and plasma norepinephrine (all *p* < 0.001), despite similar neuro-hormonal antagonism therapy. Patients with HFmrEF, on the other hand, had greater LA volume when compared to NB (*p* < 0.05), while patients with HFpEF had higher LV filling pressures and increased plasma levels of norepinephrine and NT-proBNP (all *p* < 0.05) (Supplementary Tables 1, 2).

When comparing patients with CA limited to the nighttime period with those showing CA both during the daytime and nighttime (using a cutpoint of AHI≥15 events/h) (Table 6), the latter showed worse symptoms (higher NYHA class), greater diastolic dysfunction and higher NT-proBNP in HFrEF patients (all *p* < 0.05). Patients with CA during the day and the night also showed higher norepinephrine plasma levels and worse oxygen saturation profile, as expressed by a higher time spent with a SaO_2_ below 90% (T90), both in patients with HFrEF and HFpEF (all *p* < 0.05). No significant difference was observed in patients with HFmrEF (all *p* > 0.05).

Analysis of clinical correlates of OA was only limited at nighttime given their low prevalence at daytime (Supplementary Table 3). When compared to NB, patients with nighttime OA had higher prevalence of comorbidities (obesity and hypertension in HFrEF, hypertension in HFpEF; all *p* < 0.05) and echocardiographic abnormalities (higher LA volume in HFrEF and higher sPAP in HFmrEF; all *p* < 0.05), despite a similar degree of adrenergic activation (all *p* > 0.05).

## Discussion

To our knowledge, this is the first study to address the prevalence, clinical predictors and clinical correlates of both OA and CA throughout the entire 24-h period and across the whole spectrum of HF. Patients with HFrEF had higher prevalence of CA than patients with HFmrEF and HFpEF, while OA were more common in patients with HFpEF. In HFrEF patients, specific predictors of CA and OA were identified (male gender and BMI for nighttime CA and OA; age, NYHA class and diastolic dysfunction for daytime CA), while no predictors were found in the HFmrEF and HFpEF populations (besides sPAP as predictor for CA in patients with HFmrEF). When compared to NB, patients with CA had higher sympathetic activation (as demonstrated by increased plasmatic norepinephrine levels), greater NT-proBNP levels and increased indexes of hemodynamic overload in each HF subgroups (i.e., higher LA volume and worse diastolic function in HFrEF, increased LA volume in HFmrEF and worse diastolic function in HFpEF). On the other hand, OA patients were more comorbid (in patients with HFrEF and HFpEF), had a worse hemodynamic profile (increased LA volume and sPAP in HFrEF and HFmrEF, respectively), but similar degree of neuro-hormonal activation as compared to patients with NB.

In the HFrEF population, the prevalence and prognostic significance of CA has been largely established both during the day and at night (2, 8, 25–27). In particular, patients presenting with CA throughout the 24-h (including the daytime) were shown to have an increased risk of mortality when compared with patients presenting with CA only at nighttime (2). In our study, approximately two thirds of patients presented with CA during the night, while CA were observed in about half of the population at daytime (using a AHI threshold of 5 events/h). Higher prevalence of nighttime CA over daytime CA, as observed in the current study, can be related to the loss of cortical influences and predominance of feed-back control on respiration at night, as well as to rostral fluid shift occurring in the recumbent position typical of patients with HF (28–30).

Daytime CA has been previously described in smaller studies performed in systolic HF patients with short term recordings (10 or 20 min) (5, 6), finding a prevalence ranging from 38 to 59%. Those preliminary findings were later confirmed with 24-h portable systems first in a smaller population by Brack et al. (10), and then in a larger population by our group (2). Those studies also highlighted that patients with both nighttime and daytime CA have the highest risk of experiencing life-threatening events (2, 10). In our study, HFrEF patients with CA (moderate-severe, AHI>15 events/h) throughout the 24-h (at daytime and nighttime) showed increased neuro-hormonal activation and worse diastolic and SaO_2_ profiles (higher T90, an independent prognostic marker) (7) than patients with CA occurring only at nighttime, thus providing pathophysiological insight to previous prognostic observations (2, 7). Higher T90 and norepinephrine plasma levels were found also in HFpEF patients with CA over the 24-h, suggesting that this subset of patients might be at increased risk of mortality, as already documented in HFrEF.

Of note, a greater diastolic impairment was found to be predictive of daytime CA in our study at least in HFrEF patients. Increased ventricular stiffening and impaired relaxation, together with increased LV and LA pressure, might in fact be responsible of chemoreflex activation via pulmonary J-receptor or C-fibers stimulation (31–33), thus promoting ventilatory instability, as already demonstrated in a canine model of LA pressure augmentation via balloon inflation (34).

In the HFrEF population, the prevalence of OA found in this study was similar to that found by Grimm and coworkers (35) while lower than other previous reports (36–38). Differences in patients' characteristics, study design (retrospective vs. prospective), apnea definition (AHI threshold) and detection (attended standard polysomnography vs. portable long-term ambulatory systems), and HF severity (i.e., greater diastolic dysfunction and/or MR) might help explain those discrepancies.

In HFpEF, on the other hand, we found that approximately one third of the patients presented with nighttime CA, while nighttime OA was generally more prevalent, being observed in one patient out of two. The higher prevalence of OA in patients with HFpEF may simply express the high prevalence of OA in the general population (>20% in subjects with over the age of 60) (39): those subjects may become patients with HFpEF over time (higher age compared to HFmrEF and HFrEF), due to OA related adrenergic overactivation, phasic hypoxia-reoxigenation driving to oxidative stress and inflammation and intra-pleural pressure swings (40). On the other hand, CA have been described at night also in patients with HFpEF (18, 25) and tend to emerge whenever the diastolic profile deteriorates (18). This also justifies the higher prevalence of nocturnal CA in patients hospitalized for HF (30%) (18–25), compared to the outpatient scenario (18%) (19), due to the increased clinical and haemodynamic stability in the latter case.

Daytime CA, on the other hand, have never been described in HFpEF. Interestingly, we found that 42% of HFpEF patients present with daytime CA, a prevalence slightly lower than the one observed in patients with HFrEF.

On the contrary, during the daytime OA prevalence is much lower (18%). This may be associated with a minor contribution of anatomical factors (such as obesity and increased fat deposition around the upper airway) as compared to haemodynamic or neural factors (worse diastolic profile with J-receptor stimulation, high loop-gain OA transforming to CA during the day) in awake and standing conditions (30–34, 41). Alternatively, reduced lung volume and increased prevalence of pulmonary and metabolic comorbidities in patients with OA can also impact on the plant (or the lung) gain during the day, thus promoting a shift from OA to CA (42).

Finally, we described CA in patients with HFmrEF both at nighttime (48%) and at daytime (43%). The apnea prevalence in HFmrEF has never been previously reported, and its prognostic significance still needs to be clarified, as it has only been addressed in mixed population of HFrEF and HFmrEF patients (patients with systolic HF) (8, 26, 38).

Nonetheless, we found that HFmrEF patients with CA have greater LA volumes, which can again suggest a potential role of backward failure with increased filling pressure, rather than decreased cardiac output, as the main mechanism leading to ventilatory instability in this setting (34, 43).

Presence of daytime CA in the HFmrEF population was associated with increased sPAP in this study. Whether increased sPAP is a cause or a consequence of CA is still unclear. However, it seems that increased sPAP could be ascribed to vasoconstriction of pulmonary vessels due to chemoreflex overactivation even in presence of only slightly depressed LVEF (44, 45). Similarly, intermittent hypoxia may directly cause adverse vascular remodeling on the long term, thus increasing average pulmonary pressures beyond dynamic variations due to alternating phases of CA and hyperventilation (44–46).

Nonetheless, both daytime and nighttime CA were associated with higher NT-proBNP and plasma norepinephrine values despite LVEF in this study, while plasmatic levels of norepinephrine and NT-proBNP activation did not differ between OA and NB, partially explaining the lower prognostic impact of OA as compared to CA in HF.

Of note, this is the first study describing CA as a 24-h phenomenon in spite of LVEF classification in HF. Therefore, screening for CA with tools assessing both daytime and nighttime apneas would be advisable. Similarly, therapeutic approaches that also challenge daytime CA seem desirable in patients with HFrEF, considering the prognostic significance of daytime apneas, (2) and they might be advisable also in patients with HFpEF, considering the clinical correlates associated with daytime CA observed in the current study.

## Study Limitations

Hypopneas were scored according to the main trend of central or obstructive events, as previously reported (24), which could lead to underestimation of misclassification of the events. However, invasive maneuvers such as transoesophageal pressure transducer or diaphragmatic electromyography are both unfeasible over the 24-h and unpractical in a large series of patients.

For similar reasons, we chose to use an unattended ambulatory system to screen for CA/OA, rather than performing a standard polysomnography. Despite losing information about cortical activity (essential to define the sleep/awake periods), the ambulatory system has the advantage of allowing a 24-h recording, with a diagnostic accuracy comparable to standard polysomnography (21, 22). This information, especially if supported by prognostic data (still to be acquired), will presumably change both diagnostic screening algorithms and the therapeutical approaches, including the daytime period at least when dealing with CA in HF.

Finally, a comprehensive evaluation of neuroreflexes is also lacking, especially when addressing pathophysiological mechanisms and prognosis of the respiratory disease. Further studies are needed to clarify their relative contribution to the pathophysiology and prognostic significance of CA/OA in the different HF phenotypes, also with the aim of developing tailored and rational treatments.

## Conclusions

This is the first study to describe the prevalence of both OA and CA during the whole 24-h period and, more importantly, across the whole HF spectrum. Both CA and OA are highly prevalent phenomena regardless of LVEF and can be detected both at nighttime and daytime, with CA prevalence increasing and OA prevalence decreasing as LV function worsens.

We also analyzed the clinical predictors and correlates of CA and OA in HF and found that CA are associated with worse hemodynamic profile (diastolic dysfunction, LA volume and sPAP), as well as worse symptoms, especially in HFrEF, while OA are associated with higher degree of comorbidities, especially obesity and hypertension. Those data suggest specific pathophysiological mechanisms underlying CA and OA in different HF categories and potentially different therapeutic targets. Interestingly, both nighttime and daytime CA were associated with worse neuro-hormonal activation, as opposed to OA, partially explaining the worse prognosis observed in patients with CA and identifying a high-risk subset in which treatment seems to be advisable to also obtain favorable effects on HF progression.

## Data Availability

The datasets generated for this study are available on motivated request to the corresponding author.

## Ethics Statement

The studies involving human participants were reviewed and approved by Comitato Etico Area Vasta Nord Ovest (CEAVNO) Toscana, Italy. The patients/participants provided their written informed consent to participate in this study.

## Author Contributions

CB, FG, PS, GV, NG, and GM contributed to data collecting and to the formation of a comprehensive database for analysis. FB and GI contributed to analysis of 24-h recordings as expert sleep technicians. CB, AG, CP, and ME contributed to the expert revision of 24-h recordings. CB, FG, PS, and AG contributed to data analysis, to manuscript writing and production of figures and tables. AG, CP, and ME contributed to manuscript revising.

### Conflict of Interest Statement

The authors declare that the research was conducted in the absence of any commercial or financial relationships that could be construed as a potential conflict of interest.

## Abbreviations

AHI: apnea hypopnea index
BMI: body mass index
CA: central apneas
CAI, central apnea index HF: heart failure
HFmrEF: heart failure with mid-range ejection fraction
HFpEF: heart failure with preserved ejection fraction
HFrEF: heart failure with reduced ejection fraction
IR: interquartile range
LA: left atrium
LV: left ventricular
LVEF: left ventricular ejection fraction
MR: mitral regurgitation
NB: normal breathers
NT-proBNP: N-terminal fraction of pro-B-type natriuretic peptide
NYHA: New York Heart Association
OA: obstructive apneas
OAI: obstructive apnea index
SaO_2_: oxygen saturation
T90: time spent with an oxygen saturation below 90%.
