# Supplementary material for: Central and Obstructive Apneas in Heart Failure With Reduced, Mid-Range and Preserved Ejection Fraction
Source: Front Cardiovasc Med. 2019 Sep 6;6:125. doi: 10.3389/fcvm.2019.00125 (PMC6742978; doi:10.3389/fcvm.2019.00125)
Supplement: Supplementary file 1 [file Data_Sheet_1.docx]

**Supplementary Tables**

Supplementary Table 1. Clinical correlates of nighttime CA across the whole HF spectrum

|  | **HFrEF** | | **HFmrEF** | | **HFpEF** | |
| --- | --- | --- | --- | --- | --- | --- |
|  | **NB** | **CA** | **NB** | **CA** | **NB** | **CA** |
| Age (years) | 57.5±16.9 | 67.1±11.6**^b^** | 63.6±1.0 | 66.9±11.7 | 65.7±10.5 | 73.0±10.1**^a^** |
| Males (%) | 52 | 82**^b^** | 52 | 88**^a^** | 48 | 70 |
| BMI (kg/m^2^) | 25.3±4.9 | 27.1±4.7**^a^** | 28.0±6.3 | 28.2±4.8 | 28.4±3.1 | 29.4±5.2 |
| NYHA class I-II/III-IV (%) | 71/29 | 63/36**^a^** | 82/18 | 78/22 | 74/26 | 64/35 |
| DCM (%) | 67 | 52**^a^** | 61 | 41 | 22 | 19 |
| ICM (%) | 22 | 49**^a^** | 39 | 55 | 26 | 17 |
| Other etiology (%) | 10 | 0**^a^** | 0 | 4 | 52 | 64 |
| **Comorbidities** |  |  |  |  |  |  |
| Atrial fibrillation (%) | 14 | 28**^a^** | 22 | 38 | 57 | 43 |
| Systemic hypertension (%) | 33 | 53**^a^** | 57 | 63 | 57 | 76 |
| Diabetes mellitus (%) | 21 | 33**^a^** | 26 | 31 | 26 | 34 |
| COPD (%) | 9 | 15 | 10 | 20 | 26 | 22 |
| Anemia (%) | 21 | 28 | 39 | 27 | 30 | 42 |
| Hb (g/dL) | 13.1±1.8 | 13.5±1.7 | 12.8±1.4 | 13.6±1.8**^a^** | 13.0±1.6 | 13.0±1.9 |
| Creatinine (mg/dL) | 1.1±0.4 | 1.3±0.5**^a^** | 1.2±0.8 | 1.2±0.4 | 1.2±0.8 | 1.1±0.3 |
| eGFR (mL/min/1.73m^2^) | 72 (51-87) | 60 (46-77) | 63 (49-87) | 67 (48-87) | 67 (45-88) | 66 (51-81) |
| TSH (µUI/mL) | 1.8 (1.2-2.6) | 1.8 (1.1-2.9) | 1.9 (0.9-3.2) | 1.4 (0.9-2.3) | 2.6 (0.9-2.9) | 2.0 (1.6-2.8) |
| C-reactive protein (mg/dL) | 0.3 (0.1-0.9) | 0.4 (0.1-0.8) | 0.2 (0.1-0.4) | 0.3 (0.1-0.9) | 0.3 (0.1-0.5) | 0.3 (0.2-1.3) |
| **Echocardiography** |  |  |  |  |  |  |
| LVEF (%) | 30.0±7.0 | 27.5±6.7**^a^** | 42.9±3.0 | 43.2±2.5 | 58.2±8.0 | 55.2±5.2 |
| LA volume (mL/m^2^) | 33.6±10.9 | 43.0±13.4**^b^** | 30.8±9.6 | 40.6±12.0**^a^** | 38.2±6.3 | 41.7±11.4 |
| Diastolic dysfunction II-III (%) | 29 | 57**^b^** | 20 | 38 | 14 | 27 |
| Moderate-severe MR (%) | 47 | 49 | 22 | 33 | 44 | 49 |
| TAPSE (mm) | 19.0±4.2 | 17.3±5.2**^a^** | 20.2±5.0 | 17.6±50 | 18.9±6.0 | 18.7±4.1 |
| sPAP (mmHg) | 41.2±11.3 | 44.7±12.8 | 32.9±6.0 | 42.1±14.2**^a^** | 35.7±7.4 | 39.9±10.3 |
| **Neurohormonal activation and excercise tolerance** | | | | | | |
| Hs-Troponin T (ng/L) | 17 (9-36) | 24 (14-40) | 29 (14-88) | 46 (26-83) | 15 (10-30) | 29 (13-58) |
| NT-proBNP (pg/mL) | 917 (363-2635) | 1953 (811-4515)**^a^** | 471 (207-1598) | 1038 (247-2401) | 549 (335-1443) | 1640 (587-2496)**^a^** |
| Norepinephrine (pg/mL) | 343 (265-504) | 512 (324-735)**^b^** | 323 (200-441) | 404 (253-624) | 305 (163-470) | 447 (364-752)**^a^** |
| Aldosterone (pg/mL) | 155 (108-239) | 123 (75-200)**^a^** | 115 (68-255) | 122 (73-213) | 114 (43-309) | 141 (47-180) |
| PRA (ng/mL/h) | 1.6 (0.5-4.7) | 1.1 (0.3-3.1) | 0.5 (0.2-1.4) | 0.6 (0.2-1.6) | 1.1 (0.2-3.1) | 0.3 (0.2-0.7) |
| Peak VO_2_ (%) | 48.6±20.3 | 54.6±16.1 | 70.9±11.1 | 62.9±15.8 | 69.5±21.9 | 54.6±15.6 |
| VO_2_/Kg/min (mL/Kg/min) | 16.0±7.9 | 14.0±4.7 | 18.8±4.9 | 16.6±4.4 | 17.9±11.8 | 13.4±3.9 |
| VE/VCO_2_ slope | 31.6±10.4 | 34.8±8.3**^a^** | 28.1±7.0 | 30.6±6.5 | 31.4±7.2 | 33.6±5.9 |
| SaO_2_ min (%) | 83.8±8.3 | 83.1±9.4**^a^** | 86.7±4.3 | 82.8±7.4 | 86.4±3.8 | 82.0±5.5 |
| T90 (min) | 2 (1-10) | 7 (2-14) | 5 (3-7) | 8 (2-16) | 2 (1-4) | 11 (6-15) |
| **Therapy** |  |  |  |  |  |  |
| β-blockers (%) | 91 | 96 | 96 | 94 | 77 | 76 |
| ACEi/ARB (%) | 93 | 93 | 87 | 88 | 73 | 69 |
| MRA (%) | 88 | 83 | 56 | 51 | 32 | 48 |
| Furosemide (%) | 51 | 47 | 22 | 47 | 66 | 58 |
| ICD/CRT-D (%) | 52 | 65 | 13 | 8 | 14 | 16 |

ACEi: angiotensin-converting enzyme inhibitors; ARB: angiotensin II receptor blockers; BMI: body mass index; COPD: chronic obstructive pulmonary disease; CRT-D: cardiac resynchronization therapy with defibrillator; DCM: dilated cardiomyopathy; eGFR: estimated glomerular filtration rate (with the Modification of Diet in Renal Disease-MDRD formula); Hb: hemoglobin; Hs: high sensitive; ICD: implantable cardioverter defibrillator; ICM: ischemic cardiomyopathy; LA: left atrium; LVEF: left ventricular ejection fraction; LVMI: left ventricular mass index; MR: mitral regurgitation; MRA: mineralocorticoid receptor antagonists; NT-proBNP: N-terminal fraction of pro–B-type natriuretic peptide; NYHA: New York Heart Association; PRA: plasmatic renin activity; SaO_2_:oxygen saturation; sPAP: systolic pulmonary artery pressure; T90: time spent with an oxygen saturation below 90%; TAPSE: tricuspid annular plane systolic excursion; TSH: thyroid stimulating hormone; VE/VCO_2_: ventilation to carbon dioxide production ratio; VO_2_: oxygen consumption.

Values are expressed as mean±standard deviation for variables with normal distribution, median (interquartile range) for variables with skewed distribution and percentages for categorical variables.

**^a^** p<0.05 NB vs CA; **^b^** p<0.001 NB vs CA

Supplementary Table 2. Clinical correlates of daytime CA across the whole HF spectrum

|  | **HFrEF** | | | **HFmrEF** | | | **HFpEF** | |
| --- | --- | --- | --- | --- | --- | --- | --- | --- |
|  | **NB** | **CA** | | **NB** | **CA** | | **NB** | **CA** |
| Age (years) | 60.7±14.7 | 68.6±10.3**^b^** | | 64.8±11.9 | 68.1±11.5 | | 70.9±10.1 | 71.5±9.5 |
| Males (%) | 66 | 85**^b^** | | 66 | 86**^a^** | | 47 | 76**^a^** |
| BMI (kg/m^2^) | 26.4±5.2 | 27.4±4.5**^a^** | | 29.1±3.2 | 27.9±3.6 | | 30.6±5.3 | 30.7±5.2 |
| NYHA I-II/III-IV (%) | 71/29 | 59/41**^b^** | | 84/16 | 70/30 | | 71/29 | 69/31 |
| DCM (%) | 62 | 50**^a^** | | 58 | 44 | | 14 | 13 |
| ICM (%) | 34 | 50**^a^** | | 42 | 51 | | 20 | 20 |
| Other etiology (%) | 5 | 0**^a^** | | 0 | 5 | | 66 | 66 |
| **Comorbidities** | | |  | | |  | | |
| Atrial fibrillation (%) | 18 | 30**^a^** | | 28 | 33 | | 38 | 42 |
| Systemic hypertension (%) | 43 | 54**^a^** | | 58 | 65 | | 70 | 77 |
| Diabetes mellitus (%) | 25 | 34**^a^** | | 22 | 30 | | 33 | 38 |
| COPD (%) | 12 | 16 | | 17 | 23 | | 23 | 30 |
| Anemia (%) | 35 | 28 | | 28 | 28 | | 36 | 49 |
| Hb (g/dL) | 13.3±1.7 | 13.5±1.7 | | 13.3±1.5 | 13.5±1.9 | | 12.8±1.8 | 12.9±1.9 |
| Creatinine (mg/dL) | 1.1±0.4 | 1.3±0.5**^b^** | | 1.1±0.6 | 1.2±0.6 | | 1.1±0.6 | 1.1±0.4 |
| eGFR (mL/min/1.73m^2^) | 69 (53-87) | 58 (45-72)**^b^** | | 69 (54-94) | 65 (48-82) | | 65 (47-85) | 69 (52-81) |
| TSH (µUI/mL) | 1.8 (1.3-2.8) | 1.8 (1.1-2.8) | | 1.3 (0.9-2.8) | 1.5 (0.9-2.5) | | 1.9 (1.2-3.0) | 2.1 (1.4-3.1) |
| C-reactive protein (mg/dL) | 0.3 (0.1-0.7) | 0.4 (0.1-0.9) | | 0.2 (0.1-0.4) | 0.3 (0.1-0.9) | | 0.3 (0.1-0.6) | 0.4 (0.2-1.3) |
| **Echocardiography** | | |  | | |  | | |
| LVEF (%) | 29.0±6.8 | 27.5±6.7**^a^** | | 43.5±2.8 | 43.1±2.7 | | 57.6±7.1 | 57.2±6.8 |
| LA volume (mL/m^2^) | 35.4±11.2 | 44.1±12.9**^b^** | | 32.8±8.0 | 40.0±12.8**^a^** | | 37.5±9.1 | 39.6±11.6 |
| Diastolic dysfunction II-III (%) | 27 | 63**^b^** | | 25 | 33 | | 13 | 28**^a^** |
| Moderate-severe MR (%) | 43 | 51 | | 22 | 30 | | 46 | 18 |
| TAPSE (mm) | 18.2±5.0 | 17.3±4.9 | | 19.5±5.2 | 17.9±5.1 | | 19.5±4.7 | 20.4±5.1 |
| sPAP (mmHg) | 40.7±11.4 | 44.9±12.7**^a^** | | 37.6±11.3 | 40.2±13.0 | | 40.1±13.0 | 38.8±11.6 |
| **Neurohormonal activation and excercise tolerance** | | | | | | | | |
| Hs-Troponin T (ng/L) | 20 (14-24) | 27 (15-42) | | 29 (10-46) | 46 (24-105) | | 17 (10-35) | 25 (12-52) |
| NT-proBNP (pg/mL) | 1004 (445-2495) | 2176 (950-5197)**^b^** | | 342 (140-864) | 1038 (417-2289)**^a^** | | 669 (338-1647) | 880 (330-2071)**^a^** |
| Norepinephrine (pg/mL) | 386 (250-577) | 517 (332-711)**^b^** | | 333 (215-447) | 412 (266-704)**^a^** | | 305 (210-457) | 557 (373-1144)**^f^** |
| Aldosterone (pg/mL) | 155 (97-207) | 111 (69-185)**^b^** | | 113 (89-232) | 113 (64-154) | | 100 (50-230) | 114 (46-180) |
| PRA (ng/mL/h) | 1.8 (0.6-4.2) | 0.8 (0.2-2.4)**^b^** | | 0.4 (0.2-1.5) | 0.5 (0.2-1.4) | | 0.4 (0.2-1.3) | 0.3 (0.2-1.1) |
| Peak VO_2_ (%) | 54.0±17.9 | 55.2±15.3 | | 68.0±17.7 | 65.4±17.9 | | 59.0±17.1 | 61.2±16.9 |
| VO_2_/Kg/min (mL/Kg/min) | 15.4±6.0 | 13.7±4.2 | | 17.4±5.0 | 17.2±5.8 | | 13.9±6.9 | 14.2±4.2 |
| VE/VCO_2_ slope | 32.3±8.4 | 34.6±8.3**^a^** | | 29.7±6.9 | 30.2±6.8 | | 35.6±8.4 | 32.9±6.9 |
| SaO_2_ min (%) | 86.2±8.5 | 81.7±11.7**^b^** | | 85.4±5.3 | 82.8±7.3 | | 84.5±5.0 | 80.4±5.2**^b^** |
| T90 (min) | 4 (1-11) | 8 (2-15)**^a^** | | 3 (2-8) | 8 (2-15) | | 6 (2-13) | 13 (8-19)**^b^** |
| **Therapy** |  |  | |  |  | |  |  |
| β-blockers (%) | 94 | 98 | | 96 | 95 | | 64 | 68 |
| ACEi/ARB (%) | 95 | 92 | | 88 | 88 | | 74 | 62 |
| MRA (%) | 85 | 83 | | 56 | 53 | | 38 | 39 |
| Furosemide (%) | 49 | 48 | | 34 | 44 | | 60 | 59 |
| ICD/CRT-D (%) | 59 | 64 | | 14 | 7 | | 10 | 13 |

ACEi: angiotensin-converting enzyme inhibitors; ARB: angiotensin II receptor blockers; BMI: body mass index; COPD: chronic obstructive pulmonary disease; CRT-D: cardiac resynchronization therapy with defibrillator; DCM: dilated cardiomyopathy; eGFR: estimated glomerular filtration rate (with the Modification of Diet in Renal Disease-MDRD formula); Hb: hemoglobin; Hs: high sensitive; ICD: implantable cardioverter defibrillator; ICM: ischemic cardiomyopathy; LA: left atrium; LVEF: left ventricular ejection fraction; LVMI: left ventricular mass index; MR: mitral regurgitation; MRA: mineralocorticoid receptor antagonists; NT-proBNP: N-terminal fraction of pro–B-type natriuretic peptide; NYHA: New York Heart Association; PRA: plasmatic renin activity; SaO_2_: oxygen saturation; sPAP: systolic pulmonary artery pressure; T90: time spent with an oxygen saturation below 90%; TAPSE: tricuspid annular plane systolic excursion; TSH: thyroid stimulating hormone; VE/VCO_2_: ventilation to carbon dioxide production ratio; VO_2_: oxygen consumption.

Values are expressed as mean±standard deviation for variables with normal distribution, median (interquartile range) for variables with skewed distribution and percentages for categorical variables.

**^a^** p<0.05 NB vs CA; **^b^** p<0.001 NB vs CA

Supplementary Table 3. Clinical correlates of nighttime OA across the whole HF spectrum

|  | HFrEF | | HFmrEF | | HFpEF | |
| --- | --- | --- | --- | --- | --- | --- |
|  | **NB** | **OA** | **NB** | **OA** | **NB** | **OA** |
| Age (years) | 57.5±16.9 | 64.9±11.4**^a^** | 63.6±1.0 | 71.1±10.8**^a^** | 65.7±10.5 | 71.6±9.1 |
| Males (%) | 52 | 80**^b^** | 52 | 76 | 48 | 67 |
| BMI (kg/m^2^) | 25.3±4.9 | 28.8±6.0**^b^** | 28.0±6.3 | 29.7±4.9 | 28.4±3.1 | 33.4±10.6 |
| NYHA I-II/III-IV (%) | 71/29 | 59/41**^a^** | 82/18 | 69/31 | 74/26 | 71/29 |
| DCM (%) | 67 | 52**^a^** | 61 | 59 | 22 | 5**^a^** |
| ICM (%) | 22 | 46**^a^** | 39 | 38 | 26 | 16**^a^** |
| Other etiology (%) | 10 | 1**^a^** | 0 | 3 | 52 | 78**^a^** |
| Comorbidities |  |  |  |  |  |  |
| Atrial fibrillation (%) | 14 | 19 | 22 | 28 | 57 | 34 |
| Systemic hypertension (%) | 33 | 55**^b^** | 56 | 69 | 56 | 80**^a^** |
| Diabetes mellitus (%) | 21 | 28 | 26 | 24 | 26 | 39 |
| COPD (%) | 9 | 18 | 10 | 25 | 26 | 30 |
| Anemia (%) | 21 | 31 | 39 | 32 | 30 | 47 |
| Hb (g/dL) | 13.1±1.8 | 13.3±1.7 | 12.8±1.4 | 13.3±1.8 | 13.0±1.6 | 12.6±1.8 |
| Creatinine (mg/dL) | 1.1±0.4 | 1.2±0.7 | 1.2±0.8 | 1.3±0.7 | 1.2±0.8 | 1.1±0.5 |
| eGFR (mL/min/1.73m^2^) | 72 (51-87) | 66 (51-81) | 63 (49-87) | 69 (49-81) | 67 (45-88) | 67 (51-83) |
| TSH (µUI/mL) | 1.8 (1.2-2.6) | 1.6 (1.1-2.6) | 1.9 (0.9-3.2) | 1.4 (0.9-2.2) | 2.6 (0.9-2.9) | 2.1 (1.3-3.1) |
| C-reactive protein (mg/dL) | 0.3 (0.1-0.9) | 0.3 (0.2-0.8) | 0.2 (0.1-0.4) | 0.3 (0.1-0.8) | 0.3 (0.1-0.5) | 0.4 (0.2-1.0)**^a^** |
| Echocardiography |  |  |  |  |  |  |
| LVEF (%) | 30.0±7.0 | 29.6±6.1 | 42.9±3.0 | 43.1±3.1 | 58.2±8.0 | 58.4±7.1 |
| LA volume (mL/m^2^) | 33.6±10.9 | 38.5±10.4**^a^** | 30.8±9.6 | 33.3±8.2 | 38.2±6.3 | 38.0±11.6 |
| Diastolic dysfunction II-III (%) | 29 | 33 | 20 | 22 | 14 | 19 |
| Moderate-severe MR (%) | 47 | 45 | 22 | 24 | 43 | 33 |
| TAPSE (mm) | 19.0±4.2 | 17.9±4.5 | 20.2±5.0 | 19.2±5.0 | 18.9±6.0 | 21.2±4.8 |
| sPAP (mmHg) | 41.2±11.3 | 39.8±11.7 | 32.9±6.0 | 41.6±11.8**^a^** | 35.7±7.4 | 40.6±14.1 |
| Neurohormonal activation and excercise tolerance | | | | | | |
| Hs-Troponin T (ng/L) | 17 (9-36) | 19 (9-23) | 29 (14-88) | 23 (8-39) | 15 (10-30) | 19 (12-42) |
| NT-proBNP (pg/mL) | 917 (363-2635) | 1090 (506-2445) | 471 (207-1598) | 398 (169-2189) | 549 (335-1443) | 640 (301-1478) |
| Norepinephrine (pg/mL) | 343 (265-504) | 397 (302-374) | 323 (200-441) | 408 (328-510) | 305 (163-470) | 370 (237-605) |
| Aldosterone (pg/mL) | 155 (108-239) | 120 (70-163)**^a^** | 115 (68-255) | 104 (89-157) | 114 (43-309) | 94 (45-246) |
| PRA (ng/mL/h) | 1.6 (0.5-4.7) | 1.0 (0.2-2.7) | 0.5 (0.2-1.4) | 0.4 (0.2-1.2) | 1.1 (0.2-3.1) | 0.4 (0.2-1.2) |
| Peak VO_2_ (%) | 48.6±20.3 | 57.4±15.1**^a^** | 70.9±11.1 | 68.6±21.6 | 69.5±21.9 | 64.0±14.5 |
| VO_2_/Kg/min (mL/Kg/min) | 16.0±7.9 | 14.5±4.2 | 18.8±4.9 | 15.9±7.0 | 17.9±11.8 | 13.1±3.5 |
| VE/VCO_2_ slope | 31.6±10.4 | 32.4±7.8 | 28.1±7.0 | 30.7±6.9 | 31.4±7.2 | 34.9±8.9 |
| SaO_2_ min (%) | 83.8±18.3 | 84.1±6.1**^a^** | 86.7±4.3 | 83.0±6.1**^a^** | 86.4±3.8 | 79.5±7.1**^b^** |
| T90 (min) | 2 (1-10) | 7 (2-14) | 5 (3-7) | 11 (4-25) | 2 (1-4) | 14 (7-21) |
| Therapy |  |  |  |  |  |  |
| β-blockers (%) | 91 | 100 | 96 | 100 | 77 | 61 |
| ACEi/ARB (%) | 93 | 96 | 87 | 90 | 73 | 68 |
| MRA (%) | 88 | 82 | 56 | 59 | 32 | 39**^a^** |
| Furosemide (%) | 51 | 51 | 22 | 41**^a^** | 65 | 64 |
| ICD/CRT-D (%) | 52 | 54 | 13 | 10 | 14 | 13 |

ACEi: angiotensin-converting enzyme inhibitors; ARB: angiotensin II receptor blockers; BMI: body mass index; COPD: chronic obstructive pulmonary disease; CRT-D: cardiac resynchronization therapy with defibrillator; DCM: dilated cardiomyopathy; eGFR: estimated glomerular filtration rate (with the Modification of Diet in Renal Disease-MDRD formula); Hb: hemoglobin; Hs: high sensitive; ICD: implantable cardioverter defibrillator; ICM: ischemic cardiomyopathy; LA: left atrium; LVEF: left ventricular ejection fraction; LVMI: left ventricular mass index; MR: mitral regurgitation; MRA: mineralocorticoid receptor antagonists; NT-proBNP: N-terminal fraction of pro–B-type natriuretic peptide; NYHA: New York Heart Association; PRA: plasmatic renin activity; SaO_2_: oxygen saturation; sPAP: systolic pulmonary artery pressure; TAPSE: tricuspid annular plane systolic excursion; T90: time spent with an oxygen saturation below 90%; TSH: thyroid stimulating hormone; VE/VCO_2_: ventilation to carbon dioxide production ratio; VO_2_: oxygen consumption.

Values are expressed as mean±standard deviation for variables with normal distribution, median (interquartile range) for variables with skewed distribution and percentages for categorical variables.

**^a^** p<0.05 NB vs OA; **^b^** p<0.001 NB vs OA
